# Supplementary material for: Quantifying and addressing the prevalence and bias of study designs in the environmental and social sciences
Source: Nat Commun. 2020 Dec 11;11:6377. doi: 10.1038/s41467-020-20142-y (PMC7733498; doi:10.1038/s41467-020-20142-y)
Supplement: Supplementary file 1 — Supplementary Information [file 41467_2020_20142_MOESM1_ESM.docx]

Supplementary information for: Christie et al. Quantifying and addressing the prevalence and bias of study designs in the environmental and social sciences.


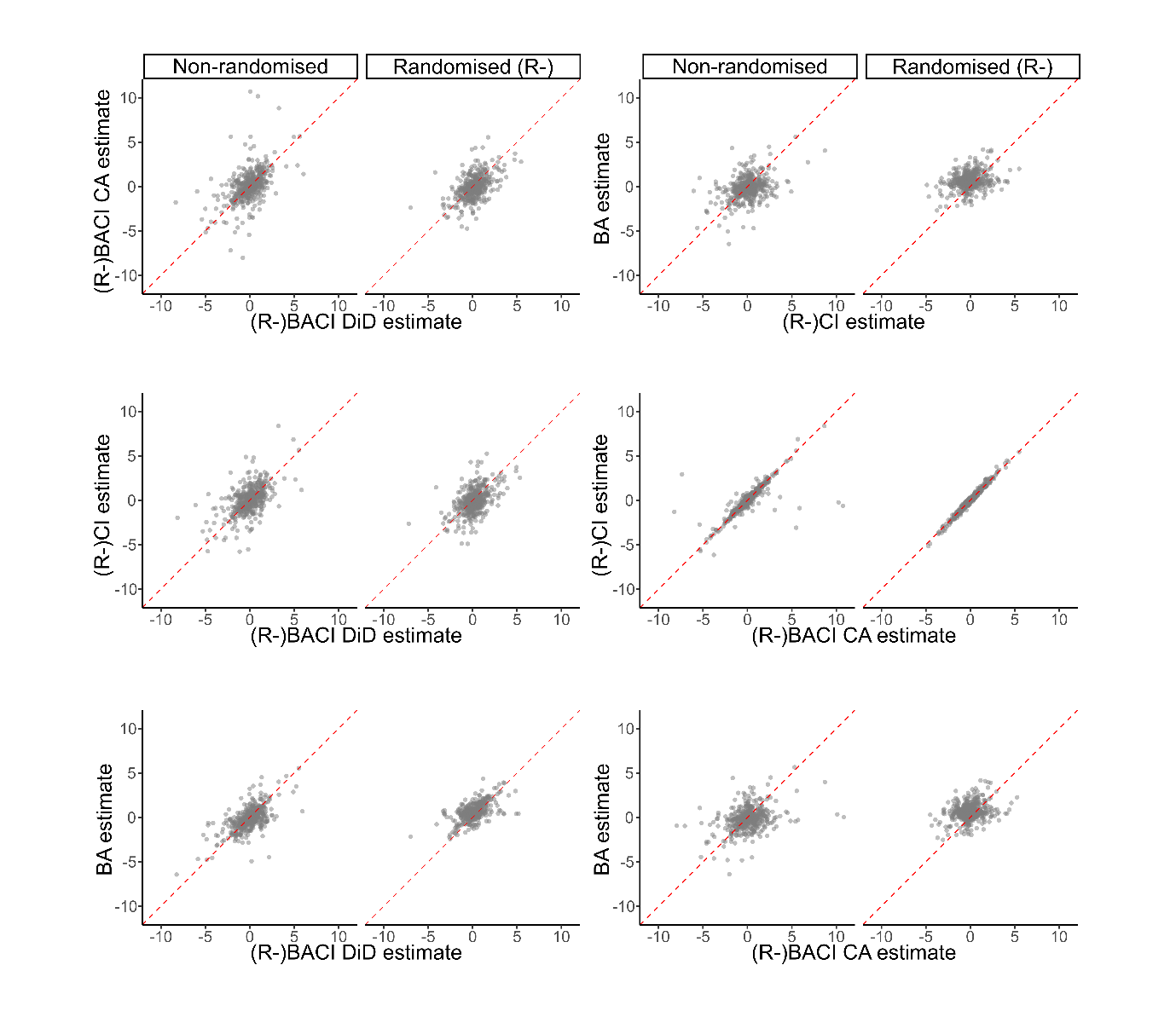
Supplementary Figure 1 – Pairwise comparisons of point estimates obtained using different study designs for 49 different datasets (non-randomised or randomised). For randomised datasets, BACI and CI axis labels refer to R-BACI and R-CI designs (denoted by ‘R-’). DiD = Difference in Differences; CA = covariance adjustment. Red lines is a 1:1 line for visualising relationship. Two extreme outliers were removed to aid data visualisation of (non-randomised) BACI CA estimates. Source data are provided as a Source Data file. BA = Before-After, CI = Control-Impact, BACI = Before-After-Control-Impact.

Supplementary Table 1 – Summary of different datasets used in within-study comparison analyses, including: the unique identifier of each dataset, the number of responses extracted and modelled from each, the number of sites and subsamples, whether randomisation was used in the collection of the original data (Y = randomised, N = non-randomised), the response measures used, and whether the impact group and control group were compared within sites (Y = within site contrast, N = between site contrast).

| Dataset ID | No. responses | No. sites | No. subsamples | Randomised | Response measure | Within site contrast |
| --- | --- | --- | --- | --- | --- | --- |
| 1 | 2 | 6 | 6 | N | Density | Y |
| 2 | 1 | 5 | 5 | N | Density | N |
| 3 | 15 | 6 | 18 | N | Count | N |
| 4 | 57 | 6 | 6 | Y | Density | Y |
| 5 | 3 | 24 | 24 | N | Density | N |
| 6 | 3 | 2 | 20 | N | Count | Y |
| 7 | 5 | 3 | 108 | N | Count | N |
| 8 | 2 | 3 | 108 | N | Count | N |
| 9 | 4 | 3 | 9 | N | Count, Density | N |
| 10 | 1 | 8 | 61 | N | Size | N |
| 11 | 6 | 20 | 20 | N | Density | Y |
| 12 | 30 | 37 | 220 | N | Count, Density | Y |
| 13 | 1 | 4 | 4 | N | Density | Y |
| 14 | 53 | 26 | 26 | N | Density | N |
| 15 | 9 | 3 | 18 | N | Density | Y |
| 16 | 37 | 3 | 35 | N | Count | N |
| 17 | 2 | 2 | 382 | N | Count | N |
| 18 | 3 | 34 | 34 | N | Count,  Density | Y |
| 19 | 28 | 6 | 6 | N | Density | Y |
| 20 | 4 | 2 | 2 | N | Density | N |
| 21 | 2 | 31 | 31 | N | Percentage | N |
| 22 | 30 | 28 | 28 | N | Percentage | N |
| 23 | 3 | 2 | 2 | N | Density | N |
| 24 | 50 | 35 | 35 | N | Count | N |
| 25 | 8 | 2 | 12 | N | Density, Size, Count | Y |
| 26 | 55 | 11 | 11 | N | Density, Count | Y |
| 27 | 21 | 1 | 1 | N | Count | Y |
| 28 | 1 | 18 | 18 | N | Count | N |
| 29 | 3 | 5 | 5 | N | Density | Y |
| 30 | 17 | 2 | 24 | N | Count | Y |
| 31 | 10 | 2 | 20 | N | Count | Y |
| 32 | 7 | 6 | 6 | N | Count | N |
| 33 | 2 | 8 | 32 | Y | Count | Y |
| 34 | 2 | 6 | 6 | Y | Density | Y |
| 35 | 13 | 4 | 467 | N | Count | Y |
| 36 | 1 | 3 | 3 | N | Count | N |
| 37 | 11 | 5 | 5 | N | Count | N |
| 38 | 2 | 4 | 4 | N | Density | Y |
| 39 | 18 | 6 | 6 | N | Density | Y |
| 40 | 29 | 3 | 3 | N | Count | N |
| 41 | 7 | 4 | 4 | N | Count | Y |
| 42 | 21 | 3 | 3 | N | Count | Y |
| 43 | 12 | 3 | 3 | N | Count | Y |
| 44 | 10 | 4 | 40 | N | Percentage | N |
| 45 | 9 | 5 | 5 | N | Density | Y |
| 46 | 1 | 3 | 18 | Y | Density | Y |
| 47 | 2 | 3 | 3 | N | Count, Size | Y |
| 48 | 2 | 4 | 4 | N | Count, Size | N |
| 49 | 421 | 19 | 57 | Y | Density | Y |
| Summary | | | | | | |
| 49 datasets | 1036 responses |  |  | Randomised:  5 datasets  Non-randomised: 44 datasets | Count: 27 datasets  Density: 19 datasets  Percentage: 3 datasets  Size: 4 datasets | Within site contrast:  27 datasets  Between site contrast:  22 datasets |

Supplementary Table 2 – Information on how different designs, and statistical methods therein, were applied to different subsets of each dataset using Generalised Linear (Mixed) Models (GL(M)Ms). DiD = Difference in Differences, CA = Covariance Adjustment. Response refers to the value of the response measure; treatment type refers to the impact or control group; time refers to the time period (before or after the impact occurred); treatment status refers to whether the site was subjected to the impact in that time period. BA = Before-After, CI = Control-Impact, BACI = Before-After-Control-Impact, R-BACI = Randomised BACI, R-CI = Randomised CI.

| Study design | Statistical method | Subset of dataset used | Fixed Effects Model structure |  |
| --- | --- | --- | --- | --- |
|  |  |  |  |  |
| BACI  R-BACI | DiD | All data | Response ~ treatment type +  time + treatment status |  |
| BACI  R-BACI | CA | All data | Post-impact within-site average ~ treatment status + pre-impact within-site average |  |
| CI R-CI | Difference | Data collected after impact (time = After) | Response ~ treatment status |  |
| BA | Difference | Impact data  (treatment type = Impact) | Response ~ treatment status |  |
