# Supplementary material for: Quantifying and addressing the prevalence and bias of study designs in the environmental and social sciences
Source: Nat Commun. 2020 Dec 11;11:6377. doi: 10.1038/s41467-020-20142-y (PMC7733498; doi:10.1038/s41467-020-20142-y)
Supplement: Supplementary file 3 — Description of Additional Supplementary Information [file 41467_2020_20142_MOESM3_ESM.pdf]

Description of Additional Supplementary Files

File Name: Supplementary Data 1

Description: References and Campbell Collaboration Coordinating Groups (i.e., wider field) for each systematic review from which data on study design prevalence was extracted.

File Name: Supplementary Data 2

Description: References of publications from which raw datasets were obtained for conducting within-study comparisons of bias in study design estimates (multiple datasets were obtained from certain studies and not all datasets were used in the final analyses – see Methods).
